# The Effect of Admission Creatinine Levels on One-Year Mortality in Acute Myocardial Infarction

**DOI:** 10.1100/2012/186495

**Published:** 2012-04-19

**Authors:** Mehmet Akif Cakar, Huseyin Gunduz, Mehmet Bulent Vatan, Ibrahim Kocayigit, Ramazan Akdemir

**Affiliations:** ^1^Deparment of Cardiology, Sakarya Education and Research Hospital, Korucuk, 54100 Sakarya, Turkey; ^2^Deparment of Cardiology, Sakarya University Medical Faculty, Sakarya, Turkey

## Abstract

*Background.* We have known that patients with renal insufficiency (creatinine level) have increased mortality for coronary artery disease. In this study, the relationship between admission creatinine level and one year mortality are evaluated in patients with acute myocardial infarction (AMI). *Method*. 160 AMI patients (127 men and 33 women with a mean age of 59 ± 13) were enrolled in the study. Serum creatinine levels were measured within 12 hours of AMI. The patients were divided into two groups according to admission serum creatinine level. (1) elevated group (serum creatinine > 1.3 mg/dL) and (2) normal group (≤1.3 mg/dL). One year mortality rates were evaluated. *Results*. Elevated serum creatinine is observed in the 27 patients (16.9%). The mean creatinine level is 1.78 ± 7 mg/dL in the elevated group and 0.9 ± 0.18 mg/dL in the normal group (*P* < 0.0001). The mortality rate of the elevated group (*n* = 7, 25.9%) is higher than that of the normal group (*n* = 9, 6.8%). A significant increase in one year mortality is also observed (*P=002*) 60. *Conclusion*. The mildly elevated admission serum creatinine levels are markedly increased to one year mortality in patients with AMI.

## 1. Introduction

Ischemic heart disease is more prevalent in patients with renal dysfunction than in the general population [1–4]. Previous studies showed an increased risk of cardiovascular disease and adverse cardiovascular outcomes in patients with renal insufficiency. End-stage renal disease, in particular, is strongly associated with the increased rate of cardiovascular diseases. There were only a few studies, though, that were focused on analysing the role of mild and moderate renal dysfunction in patients with acute myocardial infarction (AMI). Previous studies, such as GUSTO I, GUSTO II, and TIMI-2, which involved a large number of AMI patients, failed to evaluate the significance of mild and moderate renal dysfunction in morbidity and mortality [5–7]. A history of renal insufficiency or increased creatinine level on admission is associated with poor outcomes in patients with acute coronary syndrome (ACS). Recently, the Global Registry of Acute Coronary Events (GRACE) registry has proven that serum creatinine levels at admission are among the most important markers of hospital mortality in patients with ACS [8].

While renal failure greatly increases the risk of coronary disease, there have not been enough studies on the influence of mild renal impairment in patients with AMI. Thus, in this study, we aim to assess the influence of mildly elevated creatinine level in patients with AMI on one-year mortality.

## 2. Materials and Methods

We prospectively studied 160 patients (33 female and 127 male) who arrived in our hospital from January 2008 to July 2009 for acute coronary syndrome, particularly those with cases of ST and non-ST elevation MI. All patients in this study were hospitalized within 12 hours from the onset of MI symptoms and were medically treated. For patients with ST elevation, MI tissue plasminogen activator (t-PA) 100 mg was used as a fibrinolytic agent. Patients were divided into two groups according to admission serum creatinine level. The normal range of serum creatinine is between 0.6 and 1.3 mg/dL; therefore, the normal serum creatinine group was described as having ≤1.3 mg/dL (normal group) and the levels >1.3–2.0 mg/dL was described as the elevated serum creatinine group (elevated group). Acute myocardial infarction (AMI) was diagnosed on the basis of persisting chest pain associated with ST-segment elevation at two or more contiguous ECG leads. It was also diagnosed by analysing the increase in serum creatine kinase level beyond 2-fold normal upper limit. Patients were excluded if they had been admitted for more than 12 hours, had a case of valvular heart disease, had creatinine values of >2 mg/dL, or had a history of heart failure (heart failure with left ventricular ejection fraction <40%).

Clinical characteristics, such as age, gender, hypertension, diabetes, and cigarette smoking, were recorded. The presence of diabetes mellitus (DM) was considered in patients who were taking oral hypoglycemic agents, insulin or fasting plasma glucose level >126 mg/dL or >200 mg/dL at any time during hospitalisation. History of smoking and previous AMI was collected through a medical questionnaire and data from the medical chart.

Renal function was assessed by serum creatinine (Cr) measurement at admission, and glomerular filtration rate (GFR) was calculated using the Cockcroft-Gault formula.

Clinical follow-up data were obtained from outpatients' records or from a trained clinical research secretary who made telephone contacts with patients on a yearly basis. Family members of deceased patients were contacted to ascertain time of death. The primary end point of the present study was cardiac death at followup.

## 3. Statistical Analysis

Statistical analysis was performed using the Statistical Package for Social Sciences (SPSS, Chicago, Ill, USA) version 15.0 software for Windows. The data were presented as percentages for discrete variables and as means (±SD) for continuous variables. The differences in baseline characteristics were compared using the *t*-test and *χ* ^2^ test. Logistic regression analysis was performed to assess the independent value of admission creatinine, urea, glucose, and troponin levels and to estimate GFR on mortality after one-year followup (Table 3). A *P* value <0.05 was considered statistically significant.

## 4. Results

The present analysis includes a total of 160 patients (33 women and 127 men). Mean age is 59 ± 13. The number of anterior, inferior, and non-ST-segment elevation MI patients is 64 (40%), 60 (37.5%), and 36 (22.5%), respectively. Thirty-one of the patients are diabetic (19.4%). Mean hospital time of patients is 5 ± 2 days. The demographic and clinical characteristics of patients are presented in Table 1. Elevated serum creatinine was observed in the 27 patients (16.9%). The mean creatinine level was 1.78 ± 7 mg/dL in the elevated group and 0.9 ± 0.18 mg/dL in the normal group (*P* < 0.0001).

Mean GFR was 44 ± 15 mL/min in the elevated group and 96 ± 26 mL/min in the normal group (*P* < 0.0001). The mortality rate of the elevated group (*n* = 7, 25.9%) was higher than the normal group (*n* = 9, 6.8%), and a significant increase in one-year mortality was observed (*P* = 002). No significant difference was seen between the two groups in terms of the presence of the classic cardiovascular risk factors like age, smoking, diabetes mellitus, and high blood pressure. As far as AMI location is concerned, no difference was found between anterior wall, inferior wall, and non-STMI myocardial infarction. In view of the infarct localisation, seven of the dead were anterior MI, eight of them were inferior MI, and only one case was non-ST MI (*P* = 0.236). Three of the cases were diabetic, and 13 cases were nondiabetic. The clinical features of the two groups are recorded in Table 2.

The subgroup analysis of our study focuses on the age criterion of individuals. Elders under the age of 65 showed urea of 33 ± 10 mg/dL, 50 ± 20 mg/dL (*P* < 0.001), creatinine of 1 ± 0.2 mg/dL, 1.24 ± 0.6 mg/dL (*P* = 0.002), and GFR of 100 ± 28 mL/min, 63 ± 23 mL/min (*P* = 0.002). There was no reported death under age 50, but five cases of death were reported for patients between the age of 50 and 65 and 11 cases of death were reported for patients who were over 65. This shows that mortality rate is higher over the age of 65 (*P* = 0.002). Based on gender, females account to six of the deaths while males account for 10 of the deaths. The difference was not significant statistically (*P* = 0.079).

In our study, logistic regression analysis was also performed to predict the parameters that are effective on mortality. The results of these parameters are the following: creatinine (ß: 9.1, *P* = 0.002), urea: (ß: 5.6, *P* = 0.018), glucose (ß: 4.3, *P* = 0.036), troponin (ß: 9.0 *P* = 0.003), GFR (ß: 11 *P* = 0.001), and age (ß: 4.8, *P* = 0.027). These parameters showed a statistically significant impact on one-year mortality. As shown on the table, the *P* values and odds ratio of both creatinine and GFR values are more meaningful.

## 5. Discussion

It was previously shown that end-stage renal disease is a powerful predictor of death [9]. Although the precise mechanisms of the interaction between impaired renal function and coronary artery disease are not clarified, the serum creatinine concentration may be a marker for concomitant cardiovascular risk factors, such as diabetes mellitus, systemic hypertension, and advanced age. However, less severe renal dysfunction, defined by a reduction in creatinine clearance or in glomerular filtration rate (GFR) [10, 11], regardless of the underlying cause, also may lead to a significant increase in mortality after acute coronary syndromes [1, 2, 12–14].

There are multiple possible explanations for higher mortality associated with chronic kidney disease, such as specific vascular disease, combining calcified atherosclerosis and large vessel remodeling or the presence of left ventricular hypertrophy, the effect of chronic volume, or pressure overload [15–17]. Serum creatinine concentration is considered to correlate with oxidative stress, endothelial dysfunction, and progressive atherosclerosis. These biological abnormalities may increase excessive cardiovascular risk. Two factors may affect this condition. First, the higher level of serum creatinine may have an influence on the underlying clinical pathophysiological mechanisms that can lead to low cardiac output resulting in decreased renal blood flow, chronic volume overload, and diastolic left ventricular dysfunction. Second, the elevated serum creatinine group had a greater prevalence of multivessel coronary artery disease and a history of MI [18]. Comorbidities that are often associated with impaired GFR and elevated creatinine level could also explain this higher mortality rate after acute MI [16, 17].

While renal failure significantly increases coronary risk, not enough studies have been made on the influence of mild renal impairment in patients with AMI and who were medically treated on long prognosis, especially on one-year mortality. However, the early stages of kidney failure are associated with an increase in the amplitude of the pulse and high renin concentrations, which can lead to hypertrophy of the left ventricle. In addition, reductions in hemoglobin levels have been reported even with mild renal failure [19]. An association has also been found between reduction in GFR and the levels of tumour necrosis factor *α*, reactive protein C, and fibrinogen, as well as with dyslipidemia and other biomarkers [20, 21].

Reinecke et al. indicated that long-term mortality increases with higher creatinine levels, with a significant difference that starts at a level of 1.3 mg/dL and higher [22]. In the GRACE registry, an increase of 1 mg/dL in baseline Cr was associated with a 1,2-fold increase in hospital death risk. This elevation in Cr levels has been shown to have higher prognostic value than cardiac enzyme levels at admission, since present markers of myocardial necrosis are given more importance than renal function [8].

Al Suwaidi et al. reported that four ACS trial databases, including all enrolled patients, were assessed to determine 30- and 180-day outcomes. The four trials used include Global Use of Strategies to Open Occluded Coronary Arteries (GUSTO) IIb, GUSTO-III, Platelet Glycoprotein IIb/IIIa in Unstable Angina: Receptor Suppression Using Integrilin Therapy (PURSUIT) and Platelet IIb/IIIa Antagonism for the Reduction of Acute Coronary Syndrome events in a Global Organisation Network (PARAGON-A). These trials explained that mild to moderate renal abnormality independently predicts death and myocardial infarction in patients with ACS at 30-day and 6-month followup [1].

In HIJAMI Registry, Yamaguchi et al. showed that in-hospital mortality of patients with AMI was greater when the serum creatinine concentration was elevated. This was true even in patients with successful primary PCI. This adverse outcome was observed not only in patients with severe renal dysfunction but also in those with mild renal dysfunction. In this study, in-hospital mortality of patients with mild creatinine level was 17.1% (1.2–2.0 mg/dL) while those with normal creatinine level has 3.9% (<1.2 mg/dL) [23]. In addition, Gibson et al. showed that there was a stepwise increase in mortality among patients with normal, mild, or severe impaired renal function. In patients with ST-segment elevation myocardial infarction (STEMI) and who were treated with fibrinolysis, 30-day mortality was 5%, 9.5%, and 25.1%, respectively (*P* < 0.0001 for trend) [2], Unlike these studies, we excluded the patients who died in the hospital and our primary aim was to determine one-year mortality. The result was 25.9% in the elevated group and 6.8% in the normal group.

We determined that patients with mild renal dysfunction also had increased one-year mortality rate. Male gender, hypertension, smoking, infarct localisations, diabetes, left ventricle ejection fraction (LVEF), and CK-MB level were not statistically differentiated between the normal and elevated groups. Creatinine, troponin and urea level, GFR, however, were statistically differentiated, and results showed that one-year mortality was greater in the groups with higher serum creatinine levels (*P = *0.002). In addition, subgroup analysis showed higher mortality in the over 65 age group. In HIJAMI Registry, though, age was shown as an independent predictor of greater in-hospital mortality [23]. In addition, in Gibson et al.'s study, in an analysis restricted to patients 65 years old, a similar stepwise increase in mortality was observed as renal impairment increased [2]. Our result is almost identical to Gibson et al's observation in patients with AMI. Therefore, admission creatinine level may be more important in older patients.

In a new published study, Dohi et al. showed that the presence of mild renal dysfunction at ACS is associated with a worse outcome in the long term in patients with acute coronary syndrome undergoing percutaneous coronary interventions. Long-term outcomes were compared over a followup period of 1538 ± 707 days [24]. This result is similar to our data, but our patient group was different because they included non-ST elevation MI and ST elevation MI patients who were medically treated but did not undergo primary percutaneous coronary interventions. Consequently, these two studies clarify that the presence of mild renal dysfunction at ACS (both non-ST elevation and ST elevation MI patients treated with fibrinolytic agent or percutaneous coronary intervention) is associated with a worse outcome in the long term.

##  Study Limitations

First, the number of participants in the study was limited. Second, because the level of serum creatinine is of limited value in the early detection stage of renal insufficiency and is influenced by factors such as age, gender, race, and lean muscle mass, there may be a claim that the serum creatinine level is an unreliable tool in estimating renal insufficiency. This limits our study, but our findings warrant further investigation regarding the role of renal insufficiency measured by direct GFR in STEMI patients.

## 7. Conclusion

In this study, we showed that the presence of STEMI and elevated creatinine on admission is associated with increased one-year mortality, independent of other conventional risk factors. This unwanted outcome is observed not only in patients with severe renal dysfunction but also in patients with mild renal dysfunction. Creatinine levels are a cheap and fast clinical marker that can estimate long-term mortality in patients with acute coronary syndromes. Thus, AMI patients with elevated serum creatinine level on admission need close followup and intensive management for the prevention of recurrent cardiovascular events.

## Figures and Tables

**Table 1:** The general characteristics of the study patients.

Age (years)	58.94 ± 13.04
Male, *n* (%)	127 (79.4)
Hypertension, *n* (%)	38 (23.8)
Serum urea level (mg/dL)	38 ± 16
Serum creatinine level (mg/dL)	1.07 ± 0.46
Diabetes, *n* (%)	31 (19.4)
Smoking, *n* (%)	63 (39.4)
Anterior MI, *n* (%)	64 (40)
Inferior MI, *n* (%)	60 (37.5)
Non-ST MI, *n* (%)	36 (22.5)
GFR (mL/min)	87 ± 32
LVEF (%)	46 ± 9.3

MI: myocardial infarction; GFR: glomerular filtration rate; LVEF: left ventricular ejection fraction.

**Table 2:** Comparison of the characteristics of two groups according to the creatinine level.

	Creatinine ≤ 1.3 *n*: 133	Creatinine > 1.3 *n*: 27	*P* value
Anterior MI, *n* (%)	58 (36.3%)	6 (3.8%)	
Inferior MI, *n* (%)	44 (27.5%)	16 (10%)	0.032
Non ST MI, *n* (%)	31 (19.4%)	5 (3.1%)	
Diabetes, *n* (%)	23 (17.3%)	8 (29.6%)	0.139
Age (years)	65 ± 12	68 ± 12	0.094
Hypertension, *n* (%)	30 (22.6%)	8 (29.6%)	0.431
Smoking, *n* (%)	57 (42.9%)	6 (22.2%)	0.058
eGFR (mL/min)	96 ± 26	44 ± 15	<0.0001
Male, *n* (%)	109 (82%)	18 (66.7%)	0.073
Serum creatinine level (mg/dL)	0.9 ± 18	1.78 ± 7	<0.0001
CK/MB (ng/mL)	74 ± 69	98 ± 111	0.119
Troponin (ng/mL)	4.1 ± 11	28 ± 1.6	0.551
LVEF (%)	46 ± 9	47 ± 10	0.740
Serum urea level (mg/dL)	34 ± 11	59 ± 20	<0.0001
One-year mortality	9 (6.8%)	7 (25.9%)	0.002

MI: myocardial infarction; eGFR: estimated glomerular filtration rate; LVEF: left ventricular ejection fraction.

**Table 3:** Logistic regression analysis of the effect of risk factors on one-year mortality.

	Odds ratio	*P*
Serum creatinine level	9.1	0.002
Serum urea level	5.6	0.018
Admission serum glucose level	4.3	0.036
Troponin	9.0	0.003
GFR	11	0.001
Age	4.8	0.027

GFR: estimated glomerular filtration rate.

## References

[1] Al Suwaidi J, Reddan DN, Williams K (2002). Prognostic implications of abnormalities in renal function in patients with acute coronary syndromes. *Circulation*.

[2] Gibson CM, Pinto DS, Murphy SA (2003). Association of creatinine and creatinine clearance on presentation in acute myocardial infarction with subsequent mortality. *Journal of the American College of Cardiology*.

[3] Dumaine R, Collet JP, Tanguy ML (2004). Prognostic significance of renal insufficiency in patients presenting with acute coronary syndrome (the Prospective Multicenter SYCOMORE study). *American Journal of Cardiology*.

[4] Zakeri R, Freemantle N, Barnett V (2005). Relation between mild renal dysfunction and outcomes after coronary artery bypass grafting. *Circulation*.

[5] Lee KL, Woodlief LH, Topol EJ (1995). Predictors of 30-day mortality in the era of reperfusion for acute myocardial infarction: results from an international trial of 41 021 patients. *Circulation*.

[6] Topol EJ (1996). A comparison of recombinant hirudin with heparin for the treatment of acute coronary syndromes. *New England Journal of Medicine*.

[7] Morrow DA, Antman EM, Charlesworth A (2000). TIMI risk score for ST-elevation myocardial infarction: a convenient, bedside, clinical score for risk assessment at presentation: an Intravenous nPA for Treatment of Infarcting Myocardium Early II trial substudy. *Circulation*.

[8] Granger CB, Goldberg RJ, Dabbous O (2003). Predictors of Hospital Mortality in the Global Registry of Acute Coronary Events. *Archives of Internal Medicine*.

[9] Herzog CA, Ma JZ, Collins AJ (1998). Poor long-term survival after acute myocardial infarction among patients on long-term dialysis. *New England Journal of Medicine*.

[10] Levey AS, Bosch JP, Lewis JB, Greene T, Rogers N, Roth D (1999). A more accurate method to estimate glomerular filtration rate from serum creatinine: a new prediction equation. *Annals of Internal Medicine*.

[11] Levey AS, Coresh J, Bolton K (2002). K/DOQI clinical practice guidelines for chronic kidney disease: evaluation, classification, and stratification. *American Journal of Kidney Diseases*.

[12] Best PJM, Lennon R, Ting HH (2002). The impact of renal insufficiency on clinical outcomes in patients undergoing percutaneous coronary interventions. *Journal of the American College of Cardiology*.

[13] Normand SLT, Glickman ME, Sharma RGVRK, McNeil BJ (1996). Using admission characteristics to predict short-term mortality from myocardial infarction in elderly patients: results from the Cooperative Cardiovascular Project. *Journal of the American Medical Association*.

[14] Eagle KA, Lim MJ, Dabbous OH (2004). A validated prediction model for all forms of acute coronary syndrome estimating the risk of 6-month postdischarge death in an international registry. *Journal of the American Medical Association*.

[15] Sarnak MJ, Levey AS, Schoolwerth AC (2003). Kidney disease as a risk factor for development of cardiovascular disease: a statement from the American Heart Association Councils on kidney in cardiovascular disease, high blood pressure research, clinical cardiology, and epidemiology and prevention. *Hypertension*.

[16] Shlipak MG, Heidenreich PA, Noguchi H, Chertow GM, Browner WS, McClellan MB (2002). Association of renal insufficiency with treatment and outcomes after myocardial infarction in elderly patients. *Annals of Internal Medicine*.

[17] Mann JFE, Gerstein HC, Poque J, Bosch J, Yusuf S (2001). Renal insufficiency as a predictor of cardiovascular outcomes and the impact of ramipril: the HOPE randomized trial. *Annals of Internal Medicine*.

[18] Zhao L, Wang L, Zhang Y (2009). Elevated admission serum creatinine predicts poor myocardial blood flow and one-year mortality in sT-segment elevation myocardial infarction patients undergoing primary percutaneous coronary intervention. *Journal of Invasive Cardiology*.

[19] Hsu CY, McCulloch CE, Curhan GC (2002). Epidemiology of anemia associated with chronic renal insufficiency among adults in the United States: results from the Third National Health and Nutrition Examination Survey. *Journal of the American Society of Nephrology*.

[20] Lin J, Hu FB, Rimm EB, Rifai N, Curhan GC (2006). The association of serum lipids and inflammatory biomarkers with renal function in men with type II diabetes mellitus. *Kidney International*.

[21] Tonelli M, Sacks F, Pfeffer M, Jhangri GS, Curhan G (2005). Biomarkers of inflammation and progression of chronic kidney disease. *Kidney International*.

[22] Reinecke H, Trey T, Matzkies F, Fobker M, Breithardt G, Schaefer RM (2003). Grade of chronic renal failure, and acute and long-term outcome after percutaneous coronary interventions. *Kidney International*.

[23] Yamaguchi J-I, Kasanuki H, Ishii Y (2004). Prognostic significance of serum creatinine concentration for in-hospital mortality in patients with acute myocardial infarction who underwent successful primary percutaneous coronary intervention (from the Heart Institute of Japan Acute Myocardial Infarction [HIJAMI] Registry). *American Journal of Cardiology*.

[24] Dohi T, Miyauchi K, Okazaki S (2011). Long-term impact of mild chronic kidney disease in patients with acute coronary syndrome undergoing percutaneous coronary interventions. *Nephrology Dialysis Transplantation*.

